# An Immunocolorimetric Sensing System for Highly Sensitive and High-Throughput Detection of BNP with Carbon-Gold Nanocomposites Amplification

**DOI:** 10.3390/bios12080619

**Published:** 2022-08-09

**Authors:** Xin Liu, Ying Gan, Fengheng Li, Yong Qiu, Yuxiang Pan, Hao Wan, Ping Wang

**Affiliations:** 1 Biosensor National Special Laboratory, Key Laboratory for Biomedical Engineering of Education Ministry, Department of Biomedical Engineering, Zhejiang University, Hangzhou 310027, China; 2 Cancer Centre, Zhejiang University, Hangzhou 310058, China; 3 Binjiang Institute, Zhejiang University, Hangzhou 310053, China; 4 State Key Laboratory of Transducer Technology, Chinese Academy of Sciences, Shanghai 200050, China; 5 School of Biomedical Engineering, Tianjin Medical University, Tianjin 300070, China; 6 Research Institute of Intelligent Sensing, Zhejiang Lab, Hangzhou 310027, China

**Keywords:** bionic e-Eye, carbon-gold nanocomposites, immunocolorimetric sensor, BNP

## Abstract

Conventional immunocolorimetric sensing of biomolecules continues to be challenged with low sensitivity although its wide application as a diagnostic tool in medicine and biotechnology. Herein, we present a multifunctional immunocolorimetric sensing system for sensitive and high-throughput detection of B-type natriuretic peptide (BNP) with carbon-gold nanocomposite (CGNs) amplification. Using a “green” strategy, monodisperse carbon nanospheres (CNs) were successfully synthesized by glucose carbonization. A simple and efficient hydrothermal method was developed to assemble abundant gold nanoparticles (AuNPs) onto the surfaces of CNs. The resulting CGNs were characterized and utilized for biomarker detection with superior properties of easy manufacturing, good biocompatibility, satisfactory chemical stability, and high loading capacity for biomolecules. As a proof of concept, the as-prepared CGNs were conjugated with horseradish peroxidase-labeled antibody against BNP (CGNs@AntiBNP-HRP) functioning as the carrier, signal amplifier, and detector for the sensitive detection of BNP. Under optimal conditions, the established CGNs@AntiBNP-HRP immunoprobe remarkably enhanced the detection performance of BNP, achieving signal amplification of more than 9 times compared to the conventional method. Based on our self-developed bionic electronic eye (e-Eye) and CGNs@AntiBNP-HRP immunoprobe, the multifunctional sensing system displayed a wide dynamic linear range of 3.9–500 ng/mL and a LOD of 0.640 ng/mL for BNP detection with high specificity, good accuracy and reproducibility. This portable sensing system with enhanced performance demonstrates great potential for BNP detection in point of care applications, and offers a universal and reliable platform for in vitro biomarker detection.

## 1. Introduction

Precise and sensitive detection of protein biomarkers is of vital importance in clinical diagnosis and biomedical research. Accordingly, various analytical tools available for quantifying protein biomarkers have been numerously reported ranging from conventional immunocolorimetric assays to electrical sensing techniques [1,2,3]. Current electrical sensing techniques such as field-effect transistor (FET) [4,5], photoelectrochemistry (PEC) [6,7], and electrochemical sensors [8], aim for ultrahigh sensitivity and single-molecule recognition up to sub-fem to mole [9]. However, the detection reliability in practical application and high cost in the process of scaling up manufacturing hinder these electrical-based biosensors from turning into a reliable means to perform quantitative analysis. Thus, to date, the classical enzyme-linked immunosorbent assay (ELISA) remains the reliable and gold standard in detecting protein biomarkers due to the robust properties of good reliability, simple operation, acceptable sensitivity, cost efficiency, and automated high throughput [10,11].

ELISA as a high-throughput plate-based assay, allows multiple samples to be measured in a single experiment and plays a critical role in various bioanalytical settings, such as food testing [12], clinical diagnostics [13], environmental monitoring [14], and biopharmaceutical analysis [15]. Despite the wide use and desirable results of ELISA, pursuing high sensitivity and accuracy to meet specific diagnosis demands is still an ongoing endeavor. To address these challenges, the combination of nanotechnology and ELISA may serve as an effective solution to achieve improved performance for biomarker detection [13,16,17]. Gold nanoparticles (AuNPs) are most commonly used thanks to their favorable physical properties and biocompatibility, yet still suffering from limited amplification efficiency [18,19]. Therefore, great efforts have been devoted to developing other various kinds of nanomaterials for further boosting the performance of immunoassays. Among them, carbon nanospheres (CNs) gain a lot of attention because of their controllable structure, large specific surface areas, easy functionalization, favorable catalytic activity, good chemical stability, and excellent biocompatibility [20]. Most importantly, compared to other carbon nanomaterials such as graphene, fullerene, and carbon nanotubes, CNs are routinely obtained from hydrothermal carbonization of glucose without involving organic solvents, initiators, surfactants or super-high temperature such as chemical vapor deposition (CVD) [21]. Such a “green” and “convenient” method ensures the nontoxicity of the as-prepared CNs and enables their application in biochemistry and diagnostics. Notably, the surfaces of CNs feature abundant reactive oxygen functional groups that are favorable to growing plenty of nanoparticles for forming nanocomposites capable of loading high density of molecules and behaving high performance in the fields of biosensors, especially the growth of AuNPs onto CNs [22,23]. When compared to modified CNs with semiconductor quantum dots or oxide nanoparticles, AuNPs functionalized CNs (CGNs) are easy manufacturing, low cost, and environment-friendly without elaborate surface compositions, complex manipulation of the synthesis process or time-consuming steps for immobilization [21,24]. Moreover, CGNs are expected to compensate for the deficiency of limited amplification efficiency of AuNPs and present the synergistic effect of the two nanomaterials on signal amplification. As a proof of concept, we have previously prepared CGNs through a routine hydrothermal method and explored their electrochemical property in electrochemical sensors, demonstrating satisfactory results toward oxygen detection with the introduction of CGNs [25]. Herein, we further explore the performance of CGNs on the immunocolorimetric assay to extend their application.

Heart failure (HF) is the potential end-stage of almost all cardiovascular diseases (CVDs) [26]. B-type natriuretic peptide (BNP), a vital cardiac biomarker, has been acknowledged as one of the principal biomarkers of HF [27]. Individuals at risk of HF demonstrate raised serum BNP and identifying those at high risk of HF by quantifying HF biomarkers can effectively prevent premature death with appropriate intervention [28]. Although current ELISA methods for detecting BNP have been developed, most of the previous studies for ELISA-based BNP detection required large instruments with high costs such as commercial microplate readers and spectrophotometers [29]. Thus, there is an urgent need for providing more efficient and reliably in vitro diagnostic (IVD) methods and point-of-care (POC) devices in BNP clinical measurement.

**Figure 1:**
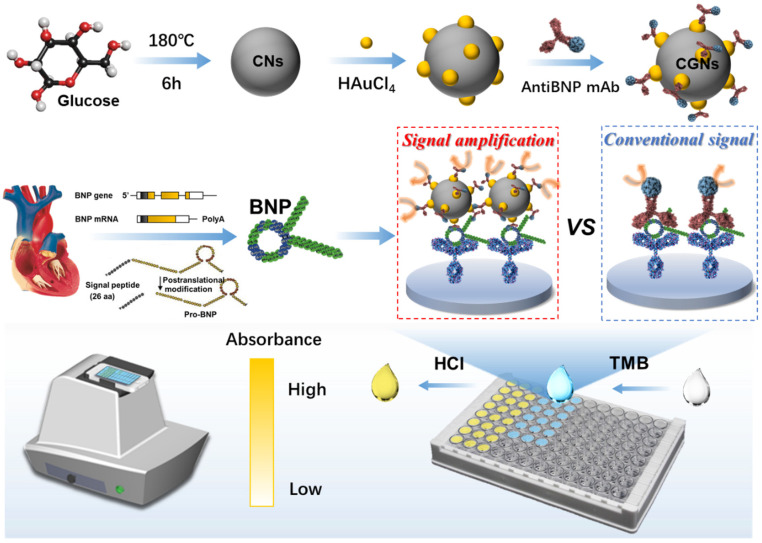
Schematic illustration of the multifunctional immunocolorimetric sensing system for sensitive detection of BNP with CGNs amplification. By immobilizing AuNPs, CGNs were successfully synthesized through hydrothermal method. Then CGNs@AntiBNP-HRP was fabricated as the carrier, signal amplifier, and detector for the sensitive detection of BNP. Combing with the portable bionic e-Eye, the sensing system could identify BNP with significantly enhanced sensitivity through the color change of antibody-antigen reactions.

In this work, a multifunctional immunocolorimetric sensing system with CGNs functioning as the carrier, signal amplifier, and detector was proposed for sensitive and high-throughput detection of BNP. CGNs were firstly synthesized through a two-step hydrothermal method, and the plentiful growth of AuNPs onto the surface of CNs provided abundant active sites for acting as efficient carriers to immobilize horseradish peroxidase-labeled antibody against BNP (CGNs@AntiBNP-HRP). In the presence of the BNP, a sandwich immunoreaction was performed utilizing CGNs@ AntiBNP-HRP as a detector. The CGNs@AntiBNP-HRP containing a quantity of HRP then catalyzed chromogenic reaction for signal amplification. A portable bionic electronic eye (e-Eye) based on a smartphone was self-developed to implement more intelligent and convenient analysis [30]. By integrating the bionic e-Eye and the multifunctional immunoprobe, the sensing system can identify BNP with significantly enhanced sensitivity through the color change of antibody-antigen reactions, which provides a universal and reliable strategy for point-of-care testing (Figure 1).

## 2. Experimental Section

### 2.1. Materials and Reagents

Recombinant human BNP protein, Anti-BNP monoclonal antibody (AntiBNP mAb), Anti-BNP polyclonal antibody labeled with HRP (AntiBNP-HRP), recombinant human troponin I (cTnI), and growth STimulation expressed gene 2 (ST2) protein, and cytokeratin-19-fragment (Cyfra21-1) were procured from Abcam (Cambridge, UK). Glucose was provided by Macklin (Shanghai, China). Bovine serum albumin (BSA), chloroauric acid (HAuCl_4_·3H_2_O), and trisodium citrate were purchased from Sigma-Aldrich (St. Louis, MI, USA). 3,3′,5,5′-Tetramethylbenzidine (TMB) was purchased from InnoReagents Co., Ltd. (Huzhou, China). Potassium carbonate (K_2_CO_3_), sodium carbonate (Na_2_CO_3_), sodium bicarbonate (NaHCO_3_), poly(diallyl dimethyl ammonium chloride) (PDDA), and all other chemicals were of analytical reagents grade and obtained from Aladdin Bio-Chem Technology Co., Ltd. (Shanghai, China). Phosphate buffer saline (PBS) used in this experiment was 0.01 M and pH 7.4. All chemicals were prepared with ultrapure water at room temperature.

### 2.2. Apparatus

Polytetrafluoroethylene (PTFE) lined hydrothermal reactor was purchased from Hongchen Instrument Equipment Co., Ltd. (Xi’an, China). The microwave method was conducted by MODE325-CEM DISCOVER microwave reactor (Matthews, North Carolina, USA). SpectraMax Paradigm microplate reader was purchased from Molecular Devices (Sunnyvale, CA, USA) and 96 well microtiter plates with high affinity were provided by Corning-Costar (New York, NY, USA). The X-ray diffraction (XRD) spectrum and X-ray photoelectron spectroscopy (XPS) was carried out using XRD-7000 diffractometer (Kyoto, Japan) and Escalab 250Xi X-ray photoelectron spectrometer (London, UK), respectively. Transmission electron microscope images were obtained by HT-770 0transmission electron microscope (Tokyo, Japan). The hydrodynamic radius and zeta-potential were collected by Malvern Zetasizer Nano-ZS (Malvern, UK).

The bionic e-Eye consists of a smartphone as a detection instrument and a portable accessory as an illumination provider. The illuminated area on the electroluminescent source is designed to place 96 well microtiter plates and the smartphone can capture the detection images in a dark hood. The obtained square image (10 × 10 pixels) of each well can be extracted from the photographed images, and the corresponding blue values (B values) are calculated for the colorimetric analysis [31].

### 2.3. Synthesis of Carbon Nanospheres

Carbon nanospheres (CNs) were synthesized before preparing the carbon-gold nanocomposites (CGNs) [21] and the procedures were as follows. Firstly, 40 mL 0.5 M glucose solution was added to a 50 mL-high-pressure reaction kettle. After reacting various times under 170 °C or 180 °C, the reaction kettle was taken out from the oven and cooled to room temperature for collecting the products. The collected products were then centrifuged at 8000 rpm for 10 min. Next, the precipitate was washed with ultra-pure water and ethanol three times after decanting the supernatant. The final precipitate was dissolved in 5 mL of ultra-pure water for the further growth of AuNPs.

### 2.4. Synthesis of Carbon-Gold Nanocomposites

In order to investigate the optimal conditions to synthesize carbon-gold nanocomposites (CGNs) with superior properties, three different methods were used to optimize the synthesis procedures of CGNs. The microwave method was first adopted and the procedures were as follows [22]. Briefly, 100 µL 1% HAuCl_4_ solution was added to 1 mL of the as-prepared CNs solution followed by the reaction in the microwave synthesizer. Parameters of the equipment for synthesizing CGNs were 250 W power, 15 min, and 100 °C. The electrostatic adsorption method was also used to synthesize the CGNs [32]. In this method, 3 mL 2% PDDA solution containing 0.02 M Tris and 0.02 M NaCl was mixed with 1 mL of CNs solution followed by stirring for 20 min. Afterward, 100 μL of the 2.3 nM fresh AuNPs with average size of 15 nm (prepared by the sodium citrate reduction of chloroauric acids and carry abundant negative charges) were added into the resultant solution with another stirring of 20 min to obtain the CGNs. Last, the hydrothermal method was employed to synthesize the CGNs based on the sodium citrate reduction [21]. 1 mL of the as-prepared CNs solution was mixed with 5 mL of 1% trisosodium citrate solution, and the automatic reflux device was used to heat the reactant until boiling. Subsequently, different volumes of 1% HAuCl_4_ solution was added with a continuously vigorous stirring for 15 min to obtain the products. The resulting products obtained from the three methods were centrifuged at 6000 rpm for 5 min to remove the free AuNPs and obtain the high purity CGNs. The precipitated CGNs were freeze-dried to store at 4 °C and could be resuspended with ultra-water for further use.

### 2.5. Synthesis of CGNs@AntiBNP-HRP Immunoprobe

To synthesize the CGNs@AntiBNP-HRP immunoprobe, 8 µL AntiBNP-HRP (0.7 mg/mL) was added into 2 mL of the as-prepared CGNs (1.1 mg) with pH 9.6 (adjusting with 0.1 M K_2_CO_3_ solution) for reacting 4 h at room temperature, followed by blocking unspecific active sites with 1% BSA and incubating for 2 h. After centrifuging at 6000 rpm for 5 min to remove excess AntiBNP-HRP, the precipitate was washed gently with PBS and then dispersed with PBS for further centrifugation and washing. After thoroughly washing and re-dispersed with 1 mL PBS containing 1% BSA (pH 7.2), CGNs labeled with AntiBNP-HRP (CGNs@AntiBNP-HRP) immunoprobe could be obtained and stored at 4 °C for further study.

### 2.6. Fabrication of GCN-Based ELISA

A 96-well microtiter plate was coated with 5 μg mL^−1^ monoclonal antibody against BNP at 4 °C overnight. After blocking with 300 μL of 2% BSA for 2 h at 37 °C, 100 μL BNP solution with various concentrations was added for incubation at 37 °C. Next, 100 μL of the AntiBNP-HRP or the CGNs@AntiBNP-HRP immunoprobe was added to incubate with BNP-contained samples. Each step was washed by PBST (PBS with 0.1% Tween-20). Finally, 100 μL TMB solution was added and incubated for 15 min for chromogenic reaction. After the addition of 100 μL 2 M HCl, different solution colors related to BNP concentration can be obtained. Combining with the microplate reader or the Bionic e-Eye, BNP concentration could be analyzed by absorbance value or image processing.

### 2.7. Serum Sample Preparation

Serum samples of healthy individuals were provided by Zhejiang University Medical College Affiliated Sir Run Run Shaw Hospital. The study protocol was approved by the institutional medical ethics review board, and all participants signed the written informed consent.

## 3. Results and Discussion

### 3.1. Optimization of CNs Synthesis

**Figure 2:**
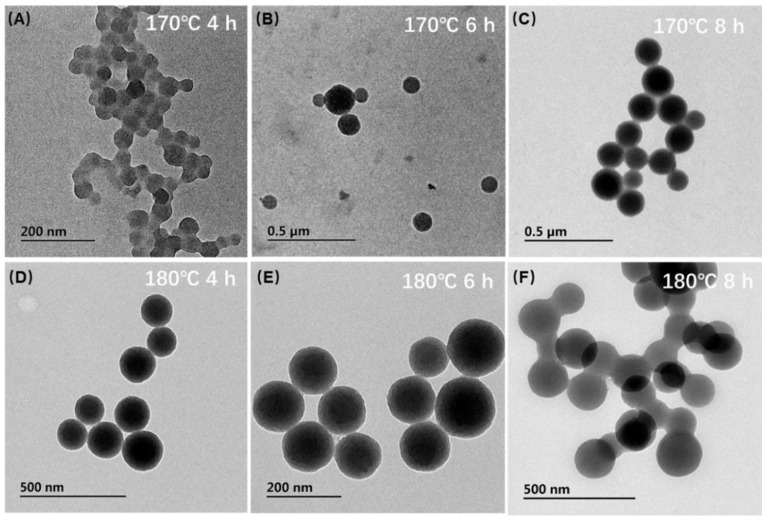
Optimization of CNs synthesis. TEM characterization of the synthesized CNs by hydrothermal method under different temperatures with various time; (**A**) 170 °C with 4 h, (**B**) 170 °C with 6 h, (**C**) 170 °C with 8 h, (**D**) 180 °C with 4 h, (**E**) 180 °C with 6 h, and (**F**) 180 °C with 8 h.

The hydrothermal method was used to synthesize the CNs, and their synthesis results at different temperatures and reaction times were investigated. As shown in the analysis result of TEM (Figure 2), the nanospheres tended to aggregate under 170 °C. However, under the temperature of 180 °C and reaction time of 6 h, we could see that CNs were distinct, and their particle size became relatively uniform with a diameter of about 130.48 ± 15.80 nm (obtained by particle size distribution of 100 particles). As a matter of the fact, the formation mechanism of CNs followed three steps. At the beginning of the reaction without sufficient time and temperature, CNs couldn’t be formed, but some aromatic compounds and oligosaccharides could be polymerized. When the heating time was long enough under high temperature and pressure, the carbonization and nucleation step would occur. This step was a crosslinking reaction caused by the intermolecular dehydration of some oligosaccharides or other macromolecules to form the carb core. With increasing reaction time, the solutes in the solution were constantly spreading to the surface of the particles so that the carb core could grow uniformly and homogeneously and finally reach the required particle size [21]. Based on the above observations, we concluded that the optimal condition to synthesize CNs was 180 °C for 6 h.

### 3.2. Growth of AuNPs on CNs (CGNs)

**Figure 3:**
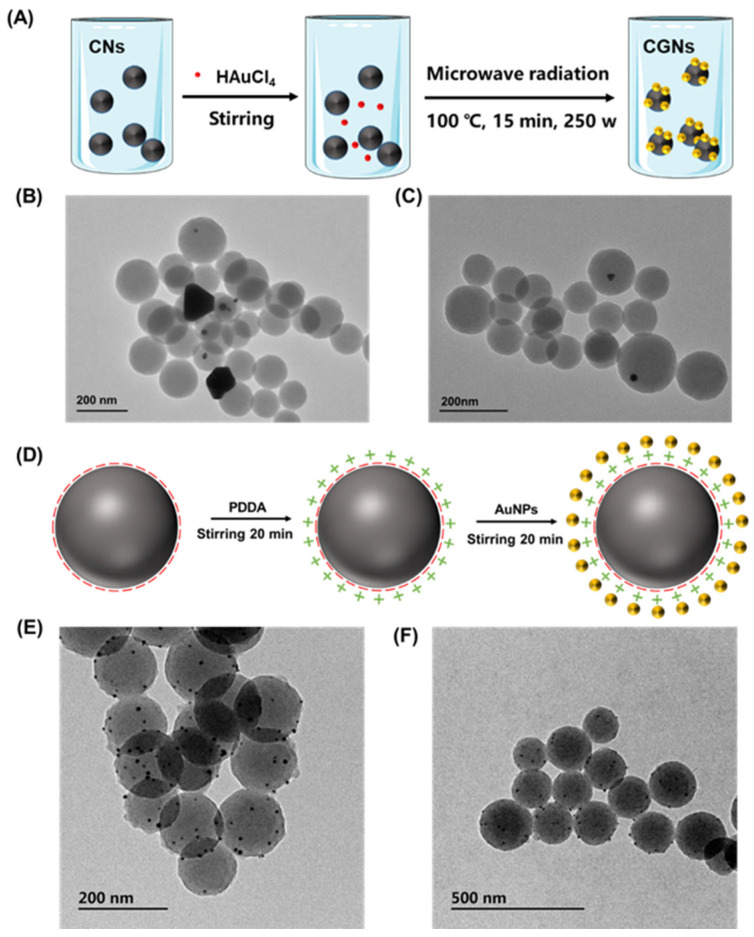
Synthesis of CGNs by microwave method and electrostatic adsorption method. (**A**) Schematic illustration of AuNPs deposited on CNs by microwave method, and (**B**,**C**) the corresponding TEM images of the synthesized CGNs. (**D**) Schematic illustration of AuNPs deposited on CNs by electrostatic adsorption method, and (**E**,**F**) the corresponding TEM characterization of the prepared CGNs.

To successfully grow AuNPs on CNs, three different modification methods were compared including the microwave method, electrostatic method, and hydrothermal method. In the microwave method, AuNPs were deposited on the surface of CNs with the help of medium heating and thermal radiation from the microwave field as shown in Figure 3A. As seen from the TEM characterization of Figure 3B,C, AuNPs could be deposited on the surface of CNs. However, only a very small part of the carbon spheres was modified with AuNPs, and a large part of them remained unmodified. Moreover, bulks of gold nanoparticles could be observed, indicating that AuNPs failed to be reduced on the surface of CNs by this method.

The second method was the electrostatic adsorption method as presented in Figure 3D. The principle of this method was mainly based on the rich negative charges existing on the surface of carbon spheres, which were aroused from their abundant functional groups such as carboxyl groups and hydroxyl groups. With the addition of PDDA, the positively charged PDDA was adsorbed onto the surface of CNs, leading to the positively charged CNs. Through electrostatic attraction, AuNPs with negative charges could be self-assembled onto the positively charged CNs, resulting in the formation of CGNs. The corresponding characterization results were shown in Figure 3E,F. It could be seen that some black gold nanoparticles were successfully deposited on the surface of CNs, and there was no obvious agglomeration of AuNPs, demonstrating a superior modification effect compared to the microwave method. Despite that, the synthesized products by the electrostatic adsorption method still suffered from less deposition of AuNPs on CNs.

**Figure 4:**
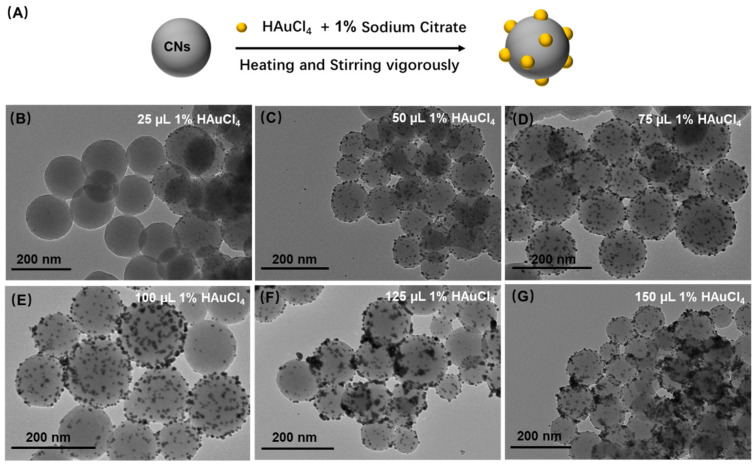
Synthesis of CGNs by hydrothermal method. (**A**) Schematic illustration of AuNPs deposited on CNs by hydrothermal method, and the corresponding TEM characterization of CGNs with different volume of 1% HAuCl_4_ of (**B**) 25 µL, (**C**) 50 µL, (**D**) 75 µL, (**E**) 100 µL, (**F**) 125 µL, and (**G**) 150 µL.

Lastly, the hydrothermal method was implemented to synthesize the CGNs as depicted in Figure 4A. The mainstay of this method was the reduction reaction between trisodium citrate and HAuCl_4_. The formed CNs normally contain abundant reactive oxygen functional groups that follow the curvature of the spheres, creating a surface with high chemical activity and good adsorption property [23]. Therefore, trisodium citrate in aqueous systems was easily spreading to the surface of CNs owing to the strong adsorption effects of CNs; conversely CNs could act as support and catalyst to promote the reduction reaction, resulting the formation of CGNs [20,21]. Hence, the optimal amount of HAuCl_4_ in the reduction reaction was studied. As presented in Figure 4, with the increasing dosage of 1% HAuCl_4_ from 25 µL to 75 µL, more AuNPs could be grown onto the surface of CNs and were gradually distributed uniformly, while AuNPs tended to accumulate on the surface of CNs after adding excessive 1% HAuCl_4_ over 75 µL. Therefore, the optimal addition amount of 1% HAuCl_4_ was 75 µL. From the morphology of the CGNs produced by the above three methods, the hydrothermal synthesis behaved well in the high-quality production of CGNs. Therefore, we adopted the hydrothermal method to modify AuNPs onto the surface of CNs.

### 3.3. Characterization of CGNs and CGNs@AntiBNP-HRP Immunoprobe

**Figure 5:**
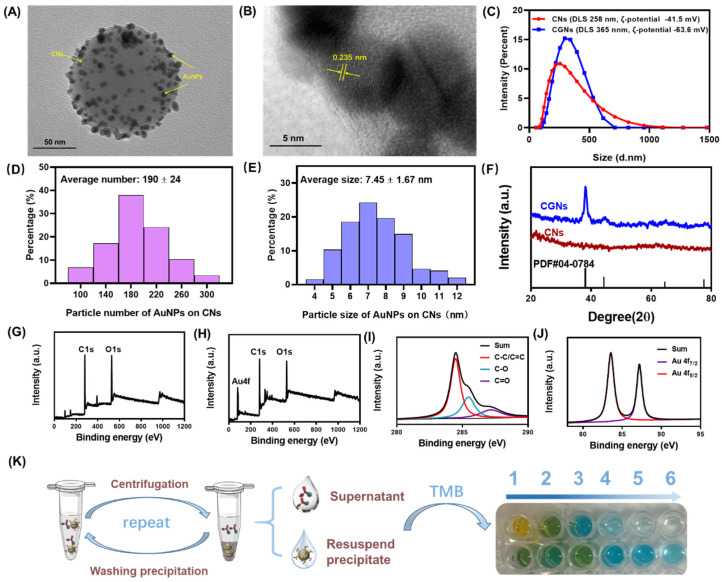
Characterization of CGNs. High resolution TEM images of (**A**) CGNs and (**B**) the crystalline structure of a gold nanoparticle on CNs with 0.235 nm lattice space. (**C**) Size distribution of CNs and CGNs by intensity. (**D**) Particle number distribution of AuNPs on CNs. (**E**) Particle size distribution of AuNPs on CNs. (**F**) XRD pattern of CNs and CGNs. XPS wide scan of (**G**) CNs and (**H**) CGNs. High-resolution XPS spectrum of (**I**) C1s of CNs and (**J**) Au4f of CGNs. (**K**) Chromogenic reaction of HRP and TMB for characterizing CGNs@AntiBNP-HRP.

Under optimal conditions of the hydrothermal method, we successfully synthesized stable and homogeneous CGNs (Figure 5A). Figure 5B shows the crystal structure of a gold nanoparticle on CNs. A lattice fringe spacing of 0.235 nm was observed, which belonged to the typical Au (111) crystal plane. Since only the gold element was introduced into the reaction during the synthesis process, it fully illustrated the expected deposition of AuNPs on CNs. Furthermore, the hydrodynamic radius and zeta-potential of CNs and CGNs were compared as shown in Figure 5C. It could be seen that the hydrodynamic radius of CNs was about 258 nm while the hydrodynamic radius of CGNs reached 365 nm because AuNPs on CNs carried a lot of negative charges resulting in larger hydrodynamic radius. Similarly, the result of zeta-potential (ζ-potential) showed that both CNs and CGNs were negatively charged and the ζ-potential of CGNs was less than CNs, which could be attributed to the successful modification of AuNPs on CNs. To estimate the number of AuNPs loaded on CNs, we drew the graph of particle number distribution of AuNPs on CNs, and as presented in Figure 5D, the average number of AuNPs particles on CNs was about 190. Meanwhile, the average particle size of AuNPs on CNs was calculated to be approximately 8 nm (Figure 5E).

In addition, XRD was used to compare the difference between CNs and CGNs. As presented in Figure 5F, there was no diffraction peak in the XRD pattern of CNs, implying their amorphous structure. However, as expected, CNs after AuNPs deposition appeared at four sharp peaks at 38.18, 44.39°, 64.57°, and 77.54° which coincided with the standard card of AuNPs (PDF#04-0784), revealing a successful synthesis of CGNs.

The XPS analysis further corroborated the synthesis of CGNs by identifying the chemical states of the elements on the surface layer. As shown in Figure 5G,H, the wide survey spectrum of CNs and CGNs exhibited binding energy of C1s and O1s, but extra Au4f could only be found on the latter which exactly stemmed from the deposited AuNPs. The presence of C and O elements in both of the CN and CGNs indicated that the reactive glucose triggering carbonization had not been completely carbonized and some oxygen-containing groups remained [22]. Figure 5I showed the high-resolution C1s spectrum of CGNs, and the peaks located at the binding energies of 284.6 eV, 286.1 eV, and 288 eV were identified as C-C, C-O, and C=O functional groups, respectively. Furthermore, the high-resolution XPS spectrum of Au4f was displayed in Figure 5J, and two sharp peaks of 83.6 eV and 87.6 eV were observed, corresponding to Au4f_7/2_ and Au4f_5/2_, respectively [33]. The above observation manifested the successful preparation of CGNs.

By taking advantage of noncovalent interactions between AuNPs on CNs and AntiBNP-HRP, we fabricated the CGNs@AntiBNP-HRP immunoprobe for signal amplification. To prove the successful preparation of the immunoprobe, we designed a reliable strategy based on the chromogenic reaction of HRP and TMB as presented in Figure 5K. More specifically, AntiBNP-HRP was directly added into the as-prepared CGNs for reacting, followed by centrifuging to remove excess free AntiBNP-HRP. Then the resultant precipitate was washed gently with PBS and further resuspended to perform another wash and centrifugation. After each centrifugation, the supernatant and part of the resuspended precipitation solution were collected and simultaneously treated with the same volume of TMB. Both the free AntiBNP-HRP in the collected supernatant and AntiBNP-HRP immobilized onto the CGNs could catalyze the oxidation of TMB and change the solution color into blue. As shown in Figure 5K, the first line was the result of color change after adding TMB to the supernatant, and the second line was the corresponding result of the resuspended solution. It could be seen after six times centrifugation, the color of the supernatant was close to colorless, and the color of the resuspended precipitation solution was still obviously blue. The colorless supernatant was a clear indicator that free AntiBNP-HRP present in the precipitate was thoroughly removed after six times wash and only CGNs@AntiBNP-HRP remained, while the visualized blue color of the resuspended precipitation solution demonstrated that plenty of AntiBNP-HRP was successfully immobilized onto the CGNs which allowed enough HRP to perform the chromogenic reaction. It could be concluded from these results that we successfully fabricated the CGNs@AntiBNP-HRP immunoprobe.

To determine the ratio of AntiBNP molecule loading on CGNs in the CGNs@AntiBNP-HRP immunoprobe, we firstly determined the input mass of AntiBNP-HRP and then calculated the mass of free AntiBNP-HRP in the supernatant by the calibration curve of AntiBNP-HRP which was obtained based on the colorimetric reaction between AntiBNP-HRP and TMB. Finally, the mass of AntiBNP-HRP (m_AntiBNP-HRP_) loading on CGNs could be calculated as 1.464 µg. As shown in Table 1, according to the input mass of CGNs (m_CGNs_), the ratio of m_AntiBNP-HRP_ to m_CGNs_ in the CGNs@AntiBNP-HRP immunoprobe was 1.331 µg/mg.

### 3.4. Optimization of Conditions for the Immunocolorimetric Probe

**Figure 6:**
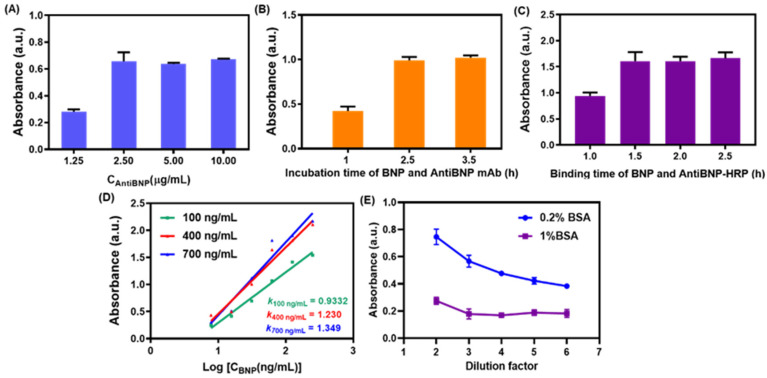
Optimization of conditions for the immunocolorimetric probe. The optimization of (**A**) the concentration of capture antibody AntiBNP mAb, (**B**) the incubation time between BNP and AntiBNP mAb, (**C**) the binding time between BNP and AntiBNP-HRP, (**D**) the linear curves between absorbance and the logarithmic value of BNP concentration (from 7.8 to 125 ng/mL) under various concentration of AntiBNP-HRP (gree line: 100 ng/mL, red line: 400 ng/mL, blue line: 700 ng/mL), and (**E**) different concentration of BSA in working buffer of CGNs@AntiBNP-HRP and various dilution factors of the buffer toward blank values.

To achieve optimally sensitive signal output, the parameters for the immunocolorimetric probe were optimized. At first, the coating concentration of capture antibody AntiBNP mAb was investigated as shown in Figure 6A. We found the absorbance ascended sharply when the concentration of AntiBNP mAb increased from 1.25 μg/mL to 2.5 μg/mL and tended to level off after 2.5 μg/mL. Therefore, 2.5 μg/mL of AntiBNP mAb was chosen as the optimal concentration for coating the ELISA plate. Immunoreaction time for this colorimetric probe was significant to improve the signal response. Figure 6B,C showed the optimal time for immunoreaction, from which we could see that the reaction time of 2.5 h ensured the equilibrium of the reaction of AntiBNP mAb with BNP, and BNP binding with AntiBNP-HRP achieved optimal response at 1.5 h. Subsequently, we investigated the dependence of sensitivity on concentration of AntiBNP-HRP by drawing the linear curves between absorbance and the logarithmic value of BNP concentration (from 7.8 to 125 ng/mL) under various concentrations of AntiBNP-HRP. As shown in Figure 6D, with the increase of AntiBNP-HRP from 100 ng/mL to 700 ng/mL, the slopes of corresponding response curves also increased from 0.9332 to 1.349. Although the results indicated a significant improvement in sensitivity as the increased input of AntiBNP-HRP [34,35], the slope varied slightly after 400 ng/mL of AntiBNP-HRP. Hence, the 400 ng/mL was selected as the optimal concentration of AntiBNP-HRP as a compromise of sensitivity and reagent costs.

One of the key ways to improve the limit of detection (LOD) of an immunocolorimetric sensor is to suppress the nonspecific adsorption as much as possible, that is to say, reducing the absorbance of a blank control group in the absence of target [34,35]. Since the nonspecific adsorption of CGNs@AntiBNP-HRP to walls of ELISA plates and AntiBNP mAb could lead to undesirable performance during the detection, we added BSA to PBS as a working buffer of the immunoprobe to prevent non-specific adsorption, and the optimal working dilution ratio of the buffer was investigated. As presented in Figure 6E, 1% BSA in the working buffer showed lower blank values compared to 0.2% BSA, revealing the relative high concentration of BSA was favorable to inhibiting the nonspecific adsorption of the immunoprobe. Therefore, PBS containing 1% BSA was selected as the working buffer to disperse CGNs@AntiBNP-HRP immunoprobe. As for the dilution ratio of PBS buffer with 1% BSA, it could be seen that with the increase of dilution ratio from 2 to 6, the blank values decreased first and remained unchanged after the dilution ratio of 3. Ultimately, 1% BSA in PBS buffer and 3-fold dilution ratio were used as the working condition of CGNs@AntiBNP-HRP immunoprobe.

### 3.5. Validation of the Immunoprobe Signal Amplification

**Figure 7:**
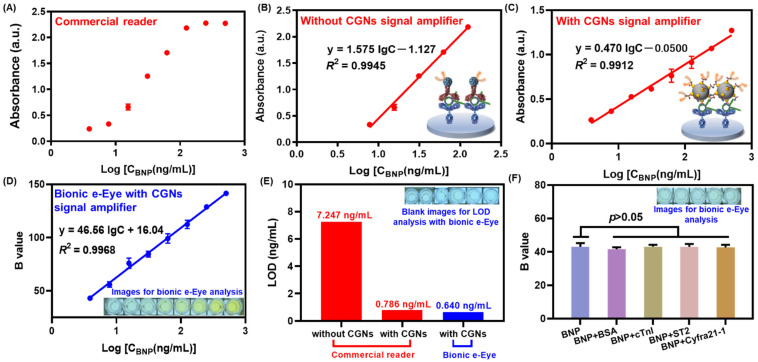
Analytical performance of the immunocolorimetric sensor based on bionic e-Eye. (**A**) The S-shaped response of BNP in different concentrations detected by the commercial microplate reader without CGNs@AntiBNP-HRP signal amplifier and (**B**) the corresponding calibration curve from 7.8–125 ng/mL; (**C**) The calibration curve of BNP from 3.9–500 ng/mL detected by the commercial microplate reader with CGNs@AntiBNP-HRP signal amplifier. (**D**) The calibration curve of BNP from 3.9–500 ng/mL detected by the bionic e-Eye with CGNs@AntiBNP-HRP signal amplifier (inset: images for bionic e-Eye analysis). (**E**) LOD comparison of the sensor with or without signal amplifier and the sensor detected using commercial microplate reader or bionic e-Eye (inset: Blank images for LOD analysis with bionic e-Eye). (**F**) The specificity of CGNs-based immunocolorimetric sensor using bionic e-Eye (inset: images for bionic e-Eye analysis). Error bars represent standard deviation (n = 3).

As a signal amplifier, the CGNs@AntiBNP-HRP immunoprobe was crucial to enhance the detection sensitivity for the target when compared to sandwich ELISA relying on conventional paired antibodies. To validate that, under optimal conditions, we simultaneously established the conventional method and CGNs@AntiBNP-HRP immunoprobe-based method for BNP detection. Figure 7A presented an S-shaped response of BNP in different concentrations detected by the commercial microplate reader, and Figure 7B showed the corresponding calibration curve from 7.8–125 ng/mL with a linear regression equation of *y* = 1.575lg*C* − 1.127 (*R*^2^ = 0.9945). The immunoprobe-based method shown in Figure 7C also obtained a good linear correlation of *y* = 0.470lg*C* − 0.05 (*R*^2^ = 0.9912) with a wider detection range of 3.9–500 ng/mL compared to the one without CGNs@AntiBNP-HRP immunoprobe as a signal amplifier. In addition, the limit of detection (LOD) in the immunoprobe-based method (Figure 7E) was calculated as 0.786 ng/mL according to the *M_b_* + 3*SD* (where *M_b_* and *SD* were the mean value and standard deviation of the blank, respectively) [34,35], which achieved more than 9 times of signal amplification than the non-probe one of 7.247 ng/mL. It was noteworthy that a smaller slope in the method with CGNs@AntiBNP-HRP immunoprobe might reveal a decreased sensitivity, which could be ascribed to the steric hindrance between immunoprobes thereby weakening the recognition to analytes. Nevertheless, we still obtained a much lower LOD and wider detection range than the non-probe one possibly due to the excellent catalytic ability of CGNs during the chromogenic reaction [25]. In summary, the above results convincingly confirm that the CGNs@AntiBNP-HRP immunoprobe has a significantly amplifying effect.

### 3.6. Analytical Performance of the Immunocolorimetric Sensor Based on Bionic e-Eye

Based on the above results, we herein combined CGNs@AntiBNP-HRP immunoprobe with our bionic e-Eye to establish a high-throughput system for the sensitive detection of BNP, and its analytical performance was evaluated by testing a series of BNP concentrations. As shown in Figure 7D, a good linear correlation between the B values and the logarithmic values of BNP levels from 3.9–500 ng/mL was obtained with an equation of *y* = 46.56lg*C* + 16.04 (*R^2^* = 0.9968), and the corresponding LOD achieved 0.640 ng/mL (Figure 7E), which behaved comparable sensitivity with the commercial one (0.786 ng/mL), which might be attributed to the superiority of the saturation channel in RGB color model of the Bionic e-Eye [30].

The specificity of the immunocolorimetric sensor was investigated in the presence of 3.9 ng/mL BNP mixed with 39 ng/mL of other interfering substances including BSA, cTnI, ST2, and Cyfra21-1. As shown in Figure 7F, the response between BNP with and without interfering substances varied slightly and showed no significant difference (*p* > 0.05), indicating the satisfactory specificity of the proposed sensor. To demonstrate the application potential of the sensor, a healthy serum sample was spiked with different concentrations of BNP (6.25 ng/mL–200 ng/mL) to conduct a recovery analysis. As Table 2 depicted, the overall recoveries were found to be 91.96–112.07%, within the variation coefficient (CV) of 1.98–14.59%, showing reliable accuracy and reproducibility in analyzing serum samples.

## 4. Conclusions

In this work, a bionic e-Eye-based high-throughput immunocolorimetric sensing system was developed for sensitively detecting BNP through CGNs@AntiBNP-HRP immunoprobe as a signal amplifier. The CGNs with abundant active sites were synthesized under the optimal conditions of the hydrothermal method and the working conditions of CGNs@AntiBNP-HRP immunoprobe were also optimized. The CGNs@AntiBNP-HRP immunoprobe was confirmed to greatly enhance the detection performance, achieving a signal amplification effect of more than 9 times compared to the non-probe method. The combination of Bionic e-Eye and high-throughput immunocolorimetric sensors not only achieved a wide dynamic range of 3.9–500 ng/mL with LOD of 0.640 ng/mL but also showed high specificity and reliable accuracy in serum samples. This work provides a universal and reliable strategy for in vitro diagnostic methods and point-of-care applications, showing promising application prospects in detecting protein biomarkers.

## Figures and Tables

**Table 1:** The ratio of m_AntiBNP-HRP_ to m_CGNs_ in the CGNs@AntiBNP-HRP immunoprobe.

Mass	AntiBNP-HRP (µg)	CGNs (mg)
m_Input_	5.6 ^a^	1.1
m_Supernatant_	4.136 ^b^	0
m_Output_	1.464	1.1
m_AntiBNP-HRP_/m_CGNs_	1.331 µg/mg	

^a^ The original concentration and input volume of AntiBNP-HRP was 0.7 mg/mL and 8 µL, respectively. Therefore, the input amount of AntiBNP-HRP was calculated as 5.6 µg. ^b^ According to the calibration curve of AntiBNP-HRP from 0.3125–5 µg/mL (y = 0.4194 *c*-0.0297, *R*^2^ = 0.9928), the amount of free AntiBNP-HRP in supernatant when preparing the immunoprobe could be determined by their absorbance, and therefore the mass of free AntiBNP-HRP could be calculated as 4.136 µg.

**Table 2:** Recovery analysis of BNP in healthy human serum sample by the proposed multifunctional immunocolorimetric sensing system.

Sample	Spike Level (ng/mL)	Found ^b^	Recovery (%) ^b^	CV (%) ^c^
Healthy human serum ^a^	200	183.91 ± 3.64	91.96 ± 1.82	1.98
	50	49.97 ± 7.29	99.94 ± 14.58	14.59
	25	24.55 ± 3.15	98.21 ± 12.61	12.84
	12.5	14.01 ± 1.36	112.07 ± 10.87	9.70
	6.25	6.86 ± 0.63	109.81 ± 10.02	9.13

^a^ The serum sample was provided by Zhejiang University Medical College Affiliated Sir Run Run Shaw Hospital. ^b^ Mean value of three measurements ± standard deviation. ^c^ CV = (SD/Mean) × 100%.

## Data Availability

No applicable.
